# Changes in health among 45–64-year-old Dutch persons before, during and after becoming unemployed or employed: a seven year follow-up study

**DOI:** 10.5271/sjweh.4016

**Published:** 2022-04-29

**Authors:** David van de Ven, Suzan JW Robroek, Karen M Oude Hengel, Alex Burdorf, Merel Schuring

**Affiliations:** 1 Erasmus University Medical Center, Department of Public Health, Rotterdam, The Netherlands; 2 Netherlands Organization for Applied Scientific Research TNO, Department of Work Health Technology, Leiden, The Netherlands

**Keywords:** body mass index, BMI, mental health, middle-aged worker, physical health, unemployment, work condition

## Abstract

**Objectives:**

This study aimed to evaluate the extent to which physical and mental health and body mass index (BMI) changed before, during and after becoming unemployed or employed, and whether these associations differ across psychosocial and physical working conditions.

**Methods:**

Participants from seven waves (2010–2017) of the Dutch longitudinal Study on Transitions in Employment, Ability, and Motivation (STREAM) aged 45–64 years were included. STREAM provides information on physical and mental health, BMI and working conditions, and was enriched with monthly information on income components from Statistics Netherlands to define employment status during 2010–2017. Annual changes in physical and mental health (0–100 scales), and BMI (kg/m^2^) before, during and after becoming unemployed (N=13 279) and employed (N=1902) were estimated with generalized linear mixed-effect models.

**Results:**

Before employed persons became unemployed they had poorer health than continuously employed persons, which worsened in the period before becoming unemployed. During the year of becoming unemployed, physical [b=1.45, 95% confidence interval (CI) 0.89–2.01] and mental health (b=1.46, 95% CI 0.85–2.07) improved, in particular among persons with unfavorable working conditions. After becoming unemployed physical health deteriorated (b=-0.52, 95% CI -0.80– -0.24) and BMI (b=0.11, 95% CI 0.03–0.19) increased, but mental health improved (b=0.33, 95% CI 0.02–0.63). Unemployed persons had better health before entering employment than continuously unemployed persons. The health of persons who entered employment did not statistically significantly change before or during the year of the transition. After entering employment, physical health deteriorated and BMI increased.

**Conclusions:**

Maintaining a healthy workforce and limiting unfavorable working conditions may contribute to the prevention of unemployment and the promotion of re-integration.

Unemployed persons have poorer physical (1) and mental health (2, 3) and are more likely to be obese (3, 4) than employed persons. The higher likelihood of poor health among unemployed persons can be explained by causation and selection mechanisms. Causation indicates that persons who become unemployed will deteriorate in health (1, 5–7), and that entering paid employment will improve health (8). Selection implies that poor health increases the risk of becoming unemployed and reduces the likelihood to enter paid employment (9, 10). Both selection and causation mechanisms may contribute to differences in health between unemployed and employed persons. Disentangling these mechanisms provides better insight into the extent to which health changes before becoming unemployed or entering paid employment and the degree to which these transitions in employment status further affect health.

Previously, researchers have studied the causal impact of unemployment on health by focusing on business closure as reason for unemployment, which limits the selection of poor health into unemployment. They showed that job loss adversely affected physical and mental health (11, 12). However, no impact was found of job loss due to business closure on body mass index (BMI) (11, 13). Other studies applied fixed-effects or hybrid models to study the impact of individual changes in employment status on changes in health outcomes (14, 15). By using these models, the authors neutralized the confounding effects of individual characteristics that remain constant over time. They showed that mental health declined after unemployment (14) and that self-reported health improved among those who entered paid employment (15). A limitation of these studies is that transitions in employment status and changes in health are compared between two consecutive time-points. It could be argued that the health effects differ depending on the time after the transition and that improvements or deteriorations in health started already before the transition, which asks for research with multiple measurements over time.

Stauder (16) used these multiple measurements over time in fixed-effects models to investigate physical and mental health immediately preceding unemployment, during the transition, and for each year following unemployment. This study showed that physical health deteriorated after becoming unemployed while the decline in mental health started already before becoming unemployed. However, only one observation before the transition to unemployment was included. In addition, the health changes of persons with a transition to unemployment were not compared with persons who remained employed during follow-up. Therefore, it remains unclear to what extent the health of persons who became unemployed developed differently compared to those continuously employed.

The impact of transitions in employment status on health outcomes might depend on the psychosocial and physical characteristics of work. A study conducted among Australian respondents of working age showed that persons with a transition from unemployment to paid employment with low job control and high psychological job demands had a greater decline in mental health than persons who remained unemployed (17). Evidence from the United Kingdom suggests that unemployed adults who obtained a job of low quality (a composite measure of low job satisfaction, low job autonomy, low job pay, and high job anxiety, high job insecurity) had poorer levels of stress-related biomarkers than persons who remained unemployed, but their mental health and physical health did not differ (18).

This study aimed to evaluate (i) the extent to which physical health, mental health and BMI changed before, during and after transitions between paid work and unemployment and (ii) whether these associations differed across psychosocial and physical working conditions. It is hypothesized that before, during and after the transition from paid employment to unemployment, physical and mental health deteriorate and BMI increases. In line with this, we hypothesize that physical and mental health improve and BMI decreases before, during, and after the transition from unemployment to paid employment – in particular in case of favorable working conditions. This study contributes to earlier research by (i) including all available observations of persons from before and after transitions in employment status, (ii) evaluating the impact of both becoming unemployed and entering paid employment, and (iii) investigating the extent to which changes in health among persons with a transition in employment status were different from those without such a transition.

## Methods

### Study design

This longitudinal study was embedded in the Dutch Study on Transitions in Employment, Ability, and Motivation (STREAM) from 2010 onwards (19). Participants aged ≥45 years filled out online questionnaires on sociodemographic factors, and characteristics with respect to work and health in the years 2010–2013, 2015–2017, and 2019. The Medical Ethical Committee of the VU University Medical Centre Amsterdam (ID: 2012–080) declared that the Medical Research Involving Human Subjects Act does not apply to STREAM. The medical ethical committee did not object to the execution of the current study. Participants were informed that their privacy would be guaranteed, that answers would be treated as confidential, and that all data would be stored on secured computer systems. For the current study, seven waves of STREAM data (2010–2013, 2015–2017) were enriched by Statistics Netherlands with monthly information from 2010 to 2017 on the main income components and social benefit pensions, derived from the Dutch tax registers and stored in the social statistical database (SSB) (20).

### Study population

The first STREAM cohort in 2010 consisted of 15 118 participants. In 2015, an additional 6738 persons participated. In total, 16 932 STREAM participants gave informed consent to link their survey data to the register-based data from Statistics Netherlands. Based on the monthly information on income components, two study samples were created to investigate the impact of transitions in employment status on health and BMI compared to those who did not change in employment status. The first sample (supplementary material, www.sjweh.fi/article/4016, figure S1) consisted of employed persons who became unemployed during follow-up and continuously employed persons (N=13 515). For the second sample (figure S2), we selected unemployed persons who entered paid work and persons who remained unemployed (N=2385). Participants aged 45–64 years old were included if they completed the STREAM survey at least once during the time that they were unemployed or employed. Participants aged ≥65 years were excluded, as this was the statutory retirement age in The Netherlands until 2013. For each participant, all STREAM observations were included from before to after the transition between unemployment and paid employment; and for those who did not change in employment status, we included the STREAM observations from the period they were unemployed or in paid work. After excluding those with missing information on all three outcomes, 13 279 participants were included in the analysis on transitions from paid work to unemployment, and 1902 participants were included in the analysis on transitions from unemployment to paid work.

### Physical health, mental health and BMI

Physical and mental health were measured with the Physical and Mental Component Summary scales obtained from the 12-item Short-Form Health Survey (SF-12) (21). These items refer to eight dimensions of health: general health, physical functioning, mental health, bodily pain, vitality, social functioning, limitations due to physical health problems, and limitations due to emotional problems. The scores on the mental and physical health scales were transformed into norm-based scores using 1998 USA standards [mean 50, standard deviation (SD) 10]. Scores could range from 0 (worst possible health status) to 100 (best possible health status). Self-reported body weight and height was used to calculate BMI and was expressed in kg/m^2^.

### Employment status

Unemployment and employment were defined based on the information on the main income components from Statistics Netherlands (20). These monthly income components can be divided into the following categories: income through paid employment, unemployment benefits, social security benefits, disability benefits, retirement benefits and economic inactivity. Persons were considered employed when they received income through paid employment as their main source of income for at least three consecutive months. Persons receiving unemployment benefits or social security benefits as their main source of income during at least three consecutive months were considered unemployed. An unemployment benefit is temporary, and level and duration depend on a person’s work history; a social security benefit is a minimum income for persons who receive insufficient household income. Persons who receive an unemployment or social security benefit have the obligation and assistance to apply for work. Self-employed persons as well as persons receiving disability benefits, retirement benefits, and economically inactive persons were not included in the current study. In case persons had multiple transitions in employment status during follow-up, only the first transition was included.

### Working conditions

Psychological job demands were assessed with four items from the Job Content Questionnaire (JCQ) on the frequency with which respondents have to work fast, perform a lot of work, work extra hard, and have hectic work (Cronbach’s alpha 0.87) (22). Autonomy included five JCQ questions about decision-making opportunities, having to think of solutions, freedom to determine the order of work, to control the work pace, and to take time off (Cronbach’s alpha 0.78) (22). Physical workload was operationalized with five questions concerning the use of extensive force during work, vibration, uncomfortable work posture, working in standing or kneeled position (Cronbach’s alpha 0.85) (23). All items were asked on a scale from 1 (always) to 5 (never). The answer categories were transformed in such a way that higher mean scores referred to more frequent exposure to unfavorable working conditions (high psychological job demands, low autonomy, high physical workload). Scale scores from 3.5 to 5 (on average always or often exposed to poor working conditions) were considered as indicative for unfavorable working conditions in the analysis.

### Statistical analysis

For persons with a transition in employment status, the closest STREAM observation before the transition date was used to determine baseline age and scores on health and BMI and for persons without a transition in employment status baseline age, health and BMI was assessed by their first observation. Working conditions observed at the time point prior to unemployment were analyzed for transitions to unemployment and, for transitions to paid employment, the working conditions observed at the time point after entering paid employment were used. For continuously employed persons, working conditions were evaluated by their first observation.

Generalized linear mixed-effects models were used to investigate changes in health and BMI before, during, and after transitions in employment status within persons and to determine differences in outcomes between persons with a transition in employment status and those without such a transition. The following regression model was used:

Y_ij_=b_0_ + b_1_A_j_ + b_2_B_i_ + b_3_C_ij_ + b_4_D_ij_ + b_5_E_ij_ + b_6_F_i_ + b_7_G_i_+ b_8_H_i_+ b_9_I_i_

In this model ‘Y_ij_’ was the dependent variable physical health, mental health or BMI for person ‘i’ at time ‘j’. In the model, a variable was included for time in years (A). A dichotomous variable was included for persons who had a transition in employment status (B=1) and those without such a transition (B=0). Two continuous variables were included for the time in years before (C) and after (D) a transition in employment status. A dichotomous variable was included to separate observations after an employment transition (E=1) from all other observations (E=0). Finally, age of persons (F), intermediate (G) and low education (H) and being male (I) were included as covariates. Additionally, a random intercept was included in the model to account for dependency across multiple observations within persons. For persons who remained employed or unemployed during follow-up, estimate b_1_ provided insight in the extent to which the outcomes changed with each year. The coefficient b_2_ indicated baseline differences in health and BMI between persons with a transition in employment status and persons without such a transition. For persons with a transition, the sum of estimates b_1_ and b_3_ reflected annual changes in health and BMI before the transition and the sum of b_1_ and b_4_ referred to annual changes in outcomes after the transition. The estimate b_3_ referred to the difference in annual change of persons before a transition in employment status compared to persons without such a transition. The coefficient b_5_ provided insight in changes in health and BMI in the year of the transition. The estimate b_6_ indicated differences in health outcomes by age, b_7_ and b_8_ respectively reflected health differences for persons with intermediate and low education compared to those with high education, and b_9_ referred to the health difference for males compared to females. Additionally, these analyses were conducted stratified by gender and the interactions between the employment transitions and gender on health and BMI were tested.

To determine whether the results were conditional upon the working conditions, additional generalized linear mixed-effects models were used with interaction terms for psychological job demands, autonomy and physical workload. For employed persons with a transition to unemployment, we estimated whether annual changes in health and BMI before, during and after the transition were different for those with unfavorable working conditions compared to neutral or favorable working conditions in their previous job. For unemployed persons with a transition to paid employment, we evaluated whether changes in health and BMI in the year of the transition and with each year following employment were different across working conditions after becoming employed.

The analyses were performed with R-Studio version 1.4.1103. The ‘lme’ function of the ‘lme4’ package was used for the generalized linear mixed-effects models with random intercepts.

## Results

### Characteristics of the study population

Table 1 shows that continuously unemployed persons were older, more often lower educated, less often male, and had poorer physical and mental health, and higher BMI than continuously employed persons and those with a transition in employment status. The exposure to high psychological job demands (35.66–38.13%) was more common than exposure to low autonomy (5.09–10.51%) or high physical workload (5.50–8.39%). For persons with a transition to unemployment on average 3.10 (SD 1.38) observations were available before the transition and 1.76 (SD 0.95) observations after the transition, whereas persons who entered paid employment had on average 1.56 (SD 0.85) observations before the transition and 2.50 (SD 1.52) observations after the transition. Continuously employed persons had on average 4.84 (SD 1.83) observations and for continuously unemployed persons on average 3.45 (SD 1.93) observations were available.

**Table 1:** Descriptive statistics of persons who became unemployed, entered paid employment or did not change their employment status during the 7-year follow-up. [SD=standard deviation; n/a=not applicable]

	Transition to unemployment (N=1503)			Continuously employed (N=11 776)			Transition to employment (N=745)			Continuously unemployed (N=1157)		
			
	Mean	SD	%	Mean	SD	%	Mean	SD	%	Mean	SD	%
Individual characteristics												
Male			55.95			55.21			54.50			49.87
Age (45–64 years)	55.77	4.71		52.83	5.47		52.96	4.63		56.39	5.63	
Education												
Low			28.74			24.76			27.92			33.10
Intermediate			39.99			39.20			43.49			39.59
High			31.27			36.04			28.59			27.31
Health outcomes												
Physical health (0–100)	51.00	8.00		51.72	7.80		52.74	7.38		46.42	12.14	
Mental Health (0–100)	50.28	9.79		52.37	7.99		51.95	8.64		49.65	10.83	
Body mass index (kg/m^2^)	27.38	4.52		26.93	4.45		27.32	4.83		27.79	5.44	
Working conditions												
Psychological job demands (1–5)	3.13	0.85		3.17	0.77		3.02	0.85		n/a	
High			37.30			38.13			35.66	n/a		
Autonomy (1–5)	2.27	0.78		2.18	0.71		2.35	0.80		n/a	
Low			8.28			5.09			10.51	n/a		
Physical workload (1–5)	1.73	0.89		1.81	0.89		1.83	0.97		n/a		
High			5.94			5.50			8.39	n/a		
Employment transitions												
Number of observations before transition (1–6)	3.10	1.38		n/a			1.56	0.85		n/a		
Number of observations after transition (1–6)	1.76	0.95		n/a			2.50	1.52		n/a		
Total number of observations	4.33	1.67		4.84	1.83		3.41	1.71		3.45	1.93	

### Transition from paid work to unemployment and changes in health outcomes

Before employed persons became unemployed, they had a 2.47 points lower mental health score (95% CI -2.93– -2.01) and 0.49 kg/m^2^ higher BMI (95% CI 0.24–0.73) compared to continuously employed persons (table 2). Persons who became unemployed also had lower physical health than continuously employed persons, although this difference was not statistically significant (b=-0.41, 95% CI -0.86–0.05). The physical health of continuously employed persons decreased by 0.19 points each year (95% CI -0.21– -0.17) and their BMI annually increased by 0.04 kg/m^2^ (95% CI 0.03–0.04), while their mental health did not change (b=-0.01, 95% CI -0.04–0.01). Persons with a transition to unemployment annually worsened in physical health (b=-0.25, 95% CI -0.37– -0.12) and mental health (b=-0.32, 95% CI -0.46– -0.18), and increased in BMI (b=0.09, 95% CI 0.05–0.13) before the transition. The annual decline in mental health and increase in BMI among persons becoming unemployed was statistically significantly larger compared to the annual changes in these outcomes among continuously employed persons. Persons who became unemployed had a 1.45 point increase in physical health (95% CI 0.89–2.01) and a 1.46 point increase in mental health (95% CI 0.85–2.07) in the year of the transition. After becoming unemployed, physical health worsened (b=-0.52, 95% CI -0.80– -0.24) and BMI increased (b=0.11, 95% CI 0.03–0.19) annually, while mental health improved (b=0.33, 95% CI 0.02–0.63) with each year. In general, the analyses for males and females showed a similar pattern. However, the annual deterioration in mental health before becoming unemployed and the annual deterioration in physical health after becoming unemployed were statistically significantly stronger for females than for males (results are not shown).

**Table 2:** Changes in physical health, mental health and body mass index (BMI) among Dutch persons aged 45–64 years in the years before, during, and after the transition from paid employment to unemployment. **Bold**: estimate is statistically significant at the 0.05 level. [CI=confidence interval.]

Transition from paid employment to unemployment	Physical health (0–100) N=13 254	Mental health (0–100) N=13 254	BMI (kg/m^2^) N=13 255
		
	b (95% CI) ^a^	b (95% CI) ^a^	b (95% CI) ^a^
Before transition			
Difference between persons becoming unemployed and continuously employed persons	-0.41 (-0.86–0.05)	**-2.47** (-2.93– -2.01)	**0.49** (0.24–0.73)
Annual change among continuously employed persons	**-0.19** (-0.21– -0.17)	-0.01 (-0.04–0.01)	**0.04** (0.03–0.04)
Annual change among persons becoming unemployed	**-0.25** (-0.37– -0.12)	**-0.32** ^b^ (-0.46– -0.18)	**0.09** ^b^ (0.05–0.13)
During transition			
Short-term change in the year of becoming unemployed	**1.45** (0.89–2.01)	**1.46** (0.85–2.07)	-0.10 (-0.26–0.06)
After transition			
Annual change among continuously employed persons	**-0.19** (-0.21– -0.17)	-0.01 (-0.04–0.01)	**0.04** (0.03–0.04)
Annual change among persons who became unemployed	**-0.52** (-0.80– -0.24)	**0.33** (0.02–0.63)	**0.11** (0.03–0.19)

a Associations are adjusted for age, gender and educational level.

b Annual change among persons becoming unemployed is significantly different compared to persons who do not change in work status at the 0.05 level.

### Transition from unemployment to paid work and changes in health outcomes

Unemployed persons had better physical (b=7.40, 95% CI 6.30–8.50) and mental health (b=3.72, 95% CI 2.65–4.79), and lower BMI (b=-0.63, 95% CI -1.15– -0.11) before they entered paid employment than those who remained unemployed (table 3). Continuously unemployed persons annually increased in mental health by 0.34 points (95% CI 0.20–0.47) and increased in BMI by 0.05 kg/m^2^ (95% CI 0.00–0.09), whereas their physical health did not change (b=-0.01, 95% CI -0.13–0.12). Before the transition from unemployment to paid work, physical (b=0.41, 95% CI -0.14–0.97) and mental health (b=0.46, 95% CI -0.16–1.09) improved and BMI decreased (b=-0.14, 95% CI -0.32–0.04) annually, although not statistically significant. During the transition to paid work, physical health (b=-0.34, 95% CI -1.22–0.53) and BMI (b=-0.10, 95% CI -0.38–0.19) decreased, while mental health increased (b=0.65, 95% CI -0.36–1.66). These changes were, however, not statistically significant. After becoming employed, physical health worsened (b=-0.64, 95% CI -0.88– -0.39) and BMI increased (b=0.11, 95% CI 0.03–0.20) with each year. Persons who entered paid employment showed a minor, but not statistically significant, annual decline in mental health (b=-0.21, 95% CI -0.50–0.08). These findings were generally similar for males and females, except that the differences in physical health and BMI between persons becoming employed end continuously unemployed persons were statistically significantly larger for females than for males (results are not shown).

**Table 3:** Changes in physical health, mental health and body mass index (BMI) among Dutch persons aged 45–64 years in the years before, during and after the transition from unemployment to paid work. **Bold**: estimate is statistically significant at the 0.05 level. [CI=confidence interval.]

Transition from unemployment to paid work	Physical health (0–100) N=1882	Mental health (0–100) N=1882	BMI (kg/m^2^) N=1885
		
	b (95% CI) ^a^	b (95% CI) ^a^	b (95% CI) ^a^
Before transition			
Difference between persons becoming employed and continuously unemployed persons	**7.40** (6.30–8.50)	**3.72** (2.65–4.79)	**-0.63** (-1.15– -0.11)
Annual change among continuously unemployed persons	-0.01 (-0.13–0.12)	**0.34** (0.20–0.47)	**0.05** (0.00–0.09)
Annual change among persons becoming employed	0.41 (-0.14–0.97)	0.46 (-0.16–1.09)	-0.14 ^b^ (-0.32–0.04)
During transition			
Short-term change in the year of becoming employed	-0.34 (-1.22–0.53)	0.65 (-0.36–1.66)	-0.10 (-0.38–0.19)
After transition			
Annual change among continuously unemployed persons	-0.01 (-0.13–0.12)	**0.34** (0.20–0.47)	**0.05** (0.00–0.09)
Annual change among persons who became employed	**-0.64** (-0.88– -0.39)	-0.21 (-0.50–0.08)	**0.11** (0.03–0.20)

a Associations are adjusted for age, gender and educational level.

b Annual change among persons becoming employed is significantly different compared to persons who do not change in work status at the 0.05 level.

### Differences across working conditions

In the years before the transition from paid work to unemployment, changes in health outcomes (table 4, supplementary tables S1 and S2) did not systematically differ across working conditions. Annual changes in physical health before becoming unemployed were not statistically significantly different for those with unfavorable working conditions compared to those with neutral/favorable working conditions (table 4). The annual deterioration in mental health before becoming unemployed was stronger for those with high psychological job demands, but did not differ for autonomy or physical workload (table S1). For BMI, persons with a high physical workload had a higher annual increase in BMI before unemployment compared to persons with a low/neutral physical workload (table S2). For the other working conditions no statistically significant interaction was found.

**Table 4:** Changes in physical health among Dutch persons aged 45–64 years in the years before, during, and after the transition from paid employment to unemployment and differences between unfavorable and favorable/neutral working conditions. **Bold**: estimate is statistically significant at the 0.05 level. [CI=confidence interval.]

Transition from paid employment to unemployment	Physical health (0-100)		

	Psychological job demands N=13 103	Autonomy N=13 123	Physical workload N=13 112
		
	b (95% CI) ^a^	b (95% CI) ^a^	b (95% CI) ^a^
Before transition			
Difference between unfavorable and favorable/neutral working conditions	**-0.96** (-1.20– -0.72)	**-1.13** (-1.66– -0.61)	**-3.35** (-3.86– -2.83)
Difference between persons becoming unemployed and continuously employed persons	-0.40 (-0.86–0.06)	-0.36 (-0.82–0.10)	-0.38 (-0.84–0.08)
Annual change among continuously employed persons	**-0.19** (-0.21– -0.16)	**-0.19** (-0.22– -0.17)	**-0.19** (-0.22– -0.17)
Annual change among persons becoming unemployed			
Unfavorable working conditions	**-0.23** (-0.41– -0.04)	-0.32 (-0.69–0.05)	-0.11 (-0.59–0.36)
Favorable/neutral working conditions	**-0.26** (-0.42– -0.11)	**-0.24** (-0.37– -0.11)	**-0.25** (-0.38– -0.12)
During transition			
Short-term change in the year of becoming unemployed			
Unfavorable working conditions	**2.54** ^b^ (1.64–3.45)	**2.71** (0.85–4.57)	**2.78** (0.46–5.10)
Favorable/neutral working conditions	**1.18** (0.49–1.87)	**1.52** (0.93–2.10)	**1.57** (0.99–2.15)
After transition			
Annual change among continuously employed persons	**-0.19** (-0.21– -0.16)	**-0.19** (-0.22– -0.17)	**-0.19** (-0.22– -0.17)
Annual change among persons who became unemployed			
Unfavorable working conditions	**-1.41** ^b^ (-1.94– -0.87)	**-1.56** ^b^ (-2.55– -0.58)	**-2.27** ^b^ (-3.62– -0.92)
Favorable/neutral working conditions	-0.26 (-0.60–0.08)	**-0.49** (-0.79– -0.20)	**-0.51** (-0.80– -0.22)

a Associations are adjusted for age, gender and educational level.

b Difference between persons with unfavorable working conditions is significantly different compared to persons with favorable or neutral working conditions at the 0.05 level.

During the transition from paid work to unemployment, physical health improved more strongly for those with unfavorable scores on each working condition (table 4). Persons with high psychological job demands (compared to persons with neutral or low psychological job demands), or low autonomy (compared to those with neutral or high autonomy) had a greater improvement in mental health in the year of the transition (table S1). The change in BMI in the year of the transition did not differ across working conditions (table S2).

After the transition to unemployment, physical health worsened more strongly each year for persons with unfavorable working conditions compared to neutral or favorable working conditions (table 4). After becoming unemployed, mental health annually improved for persons with high psychological job demands and worsened for those with low autonomy and high physical workload. These changes were however not statistically significant. Mental health improved annually for persons with favorable or neutral scores on each working condition (table S1). In addition, persons who had low autonomy had a greater annual increase in BMI after becoming unemployed than persons with neutral or high autonomy (table S2).

In the year of the transition from unemployment to paid work, changes in health outcomes did not statistically significantly differ across working conditions (table 5, supplementary tables S3 and S4). The annual decline in physical health after entering paid work was however stronger for those with high psychological and physical job demands compared to those with neutral or low demands (table 5). No statistically significant interactions with unfavorable working conditions were found for changes in mental health and BMI after entering paid employment (tables S3 and S4).

**Table 5:** Changes in physical health among Dutch persons aged 45–64 years in the years before, during, and after the transition from unemployment to paid work and differences between unfavorable and favorable/neutral working conditions. **Bold**: estimate is statistically significant at the 0.05 level. [CI=confidence interval.]

Transition from unemployment to paid work	Physical health (0-100)		

	Psychological job demands N=1877	Autonomy N=1877	Physical workload N=1877
		
	b (95% CI) ^a^	b (95% CI) ^a^	b (95% CI) ^a^
Before transition			
Difference between persons becoming employed and continuously unemployed persons	**7.37** (6.27–8.47)	**7.42** (6.32–8.52)	**7.38** (6.29–8.48)
Annual change among continuously unemployed persons	-0.01 (-0.13–0.12)	-0.00 (-0.13–0.12)	-0.01 (-0.13–0.12)
Annual change among persons becoming employed	0.36 (-0.20–0.91)	0.39 (-0.17–0.94)	0.36 (-0.20–0.92)
During transition			
Short-term change in the year of becoming employed			
Unfavorable working conditions	-0.51 (-1.79–0.77)	-0.84 (-3.04–1.36)	-0.03 (-2.46–2.39)
Favorable/neutral working conditions	-0.28 (-1.30–0.73)	-0.35 (-1.26–0.56)	-0.41 (-1.32–0.50)
After transition			
Annual change among continuously unemployed persons	-0.01 (-0.13–0.12)	-0.00 (-0.13–0.12)	-0.01 (-0.13–0.12)
Annual change among persons who became employed			
Unfavorable working conditions	**-1.12** ^b^ (-1.55– -0.70)	-0.75 (-1.73–0.23)	-1.71 ^b^ (-2.54– -0.87)
Favorable/neutral working conditions	**-0.35** (-0.66– -0.04)	**-0.61** (-0.87– -0.36)	**-0.51** (-0.77– -0.24)

a Associations are adjusted for age, gender and educational level.

b Difference between persons with unfavorable working conditions is significantly different compared to persons with favorable or neutral working conditions at the 0.05 level.

## Discussion

This study showed that employed persons had poorer physical and mental health and higher BMI before they became unemployed than persons who remained employed. Persons who became unemployed declined in physical and mental health and increased in BMI in the years before the transition. During the transition to unemployment, physical and mental health improved, in particular for those with unfavorable working conditions. After becoming unemployed, physical health declined and BMI increased annually, especially for persons with unfavorable working conditions. Mental health annually improved after becoming unemployed among those with favorable or neutral working conditions. Unemployed persons had better health and lower BMI before they entered paid employment compared to continuously unemployed persons. Before entering paid employment health annually improved and BMI decreased. In the year of entering paid employment, changes in health outcomes did not reveal a clear pattern. After becoming employed, health declined and BMI increased annually. Becoming employed was especially detrimental to physical health for workers with high psychological and physical work demands.

The findings indicated that persons with poor health were selected into unemployment and persons with good health into paid work, which is in line with our hypotheses and findings from previous studies (9, 10, 15). One other study (16) focused on changes in physical health and mental health before, during and after unemployment during a 7-year follow-up period. This study also provided support for the presence of a selection process by showing that a decline in mental health preceded unemployment. However, the author did not demonstrate a decline in physical health before unemployment. This could be explained because only one time point before unemployment was investigated, and therefore changes that occurred earlier were not taken into account.

The current study found weak support for a deterioration in health after becoming unemployed, which is in contrast to our hypotheses. Although physical health declined and BMI increased after the transition to unemployment, these changes already started before becoming unemployed. In contrast to findings from previous studies (11–14, 16), we found evidence for improvements in physical and mental health during the transition and improvement in mental health in the years following unemployment. It is argued that unemployed persons have unfulfilled manifest (income) and latent (time structure, being active, social status, collective purpose, and social contact) needs compared to employed persons, resulting in negative health effects (24, 25). However, as persons become older they might consider unemployment as an opportunity to recover from demands at work and exploit other activities, just as with retirement (26, 27), which could explain the improvements in health in our middle-aged study population (age ≥45 years). Another explanation is that health might fluctuate over the course of the follow-up period and, thus, after the deterioration in physical health and mental health before unemployment, it is more likely that health will improve rather than deteriorate.

Unlike previous studies (15, 28), we did not find a positive health impact of entering paid employment. In contrast to our hypotheses, we showed that physical health declined and BMI increased with each year after becoming employed. It could be argued that entering paid employment later in life might deteriorate health, in particular in case of unfavorable working conditions, and that the harmful effects outweigh the benefits of work. Unfavorable working conditions contribute to the decline in physical health that comes with age. As workers become older they become less physically active during leisure time (29), which increases the risk of obesity and cardiovascular diseases (30). This might explain why we found a decline physical health and increase in BMI after becoming employed, rather than positive health effects.

The current study showed that short-term improvements in health after unemployment were more pronounced for persons with unfavorable psychosocial working conditions. Since these persons are more likely to experience job strain (31), they might perceive unemployment even more as a chance to recover from work, compared to those with favorable working conditions. Likewise, Fleischmann et al (27) found that mental health of British civil servants improved in the short-term after retirement, in particular among those with poorer psychosocial working conditions. We also found that the decline in health outcomes in the years following unemployment were more profound for persons who had unfavorable working conditions. Evidence suggests that exposure to unfavorable working conditions increases the risk of having psychiatric disorders, musculoskeletal symptoms and cardiovascular diseases, and that these consequences become visible after a longer time period (31). Hence, persons could suffer from the negative consequences of exposure to unfavorable working conditions after having lost their job. Whereas a previous study points to the negative psychological impact of obtaining a job with high demands (17), our study showed that entering paid employment was especially detrimental to physical health for those exposed to high psychological and physical demands.

The main strengths of this study were the insight provided into selection and causation mechanisms concerning changes before, during and after transitions between unemployment and paid work, and the investigation of differences in associations across psychosocial and physical working conditions. Other strengths included the use of a large nationally representative sample with a 7-year follow-up period, and a combination of register data on monthly employment status and self-reported data on a variety of health outcomes and working conditions. Several limitations have to be considered as well. In the current study we were not able to exclude persons with social security benefits who were exempted from the obligation to actively search for a job. However, the vast majority of persons with social security benefits had the obligation to actively search for a job. Furthermore, individual health status is associated with the transition to unemployment. In the selection of persons who exited paid employment through unemployment, we will have an overrepresentation of persons with a poorer health. In general, a decline in health before the transition is followed by an improvement after the transition, partly attributable to regression to the mean. Therefore, we need to be careful in making statements on the causal impact of employment transitions on changes in health, in particular when interpreting the improvements in health during and after becoming unemployed. Another limitation is that the study population is older compared to other studies on the health effects of changes between paid work and unemployment. As a result, the generalizability of our findings is limited to workers aged ≥45 years. In light of the differences in findings between our study and other studies, future research could determine whether the selection and causation mechanisms are different depending on the age at which transitions take place. The last limitation is that the working conditions had a skewed distribution. In particular, most persons were never exposed to high physical workload. In this study, we used the average scores on scales for each working condition to select those with the highest psychological and physical demands. As a result, we had less statistical power to detect differences between unfavorable and neutral or favorable working conditions, especially when investigating the health impacts of entering paid employment across working conditions.

In conclusion, this study indicated that poor health preceded unemployment and might be an important barrier for becoming employed among workers aged ≥45 years. Becoming unemployed could be beneficial to health in the short-term, in particular among persons who had unfavorable psychosocial working conditions before exit from paid employment. However, in the years following unemployment, the impact of the transition varies across health outcomes and depends on working conditions. In the years following unemployment, physical health deteriorated and BMI increased especially for persons with unfavorable working conditions, while mental health improved for persons with neutral or favorable working conditions. Entering paid employment at an older age after a period of unemployment might deteriorate health, especially when working conditions are unfavorable. Health promotion initiatives may contribute to the prevention of unemployment and to support re-integration to work, and policies aimed at improving working conditions might mitigate the negative health impact of changes between unemployment and paid work.

### Funding

This study is made possible by the Netherlands Organisation for Health Research and Development (ZonMw); project number 531001403.

### Conflict of interest

None declared.

## Supplementary material

Supplementary material
