# Supplementary material for: Changes in health among 45–64-year-old Dutch persons before, during and after becoming unemployed or employed: a seven year follow-up study
Source: Scand J Work Environ Health. 2022 Apr 29;48(4):283–92. doi: 10.5271/sjweh.4016 (PMC9524163; doi:10.5271/sjweh.4016)
Supplement: Supplementary material [file SJWEH-48-283-S001.pdf]

Changes in health among 45–64-year-old Dutch persons before, during and after becoming unemployed or employed: a seven year follow-up study[1]

by David van de Ven, MSc, Suzan JW Robroek, PhD,[2] Karen M Oude Hengel, PhD, Alex Burdorf, PhD, Merel Schuring, PhD

1. *Supplementary material*
2. *Correspondence to: Suzan Robroek, Erasmus University Medical Center, Department of Public Health, P.O. Box 2040, 3000 CA Rotterdam, The Netherlands. [E-mail: s.robroek@erasmusmc.nl]*

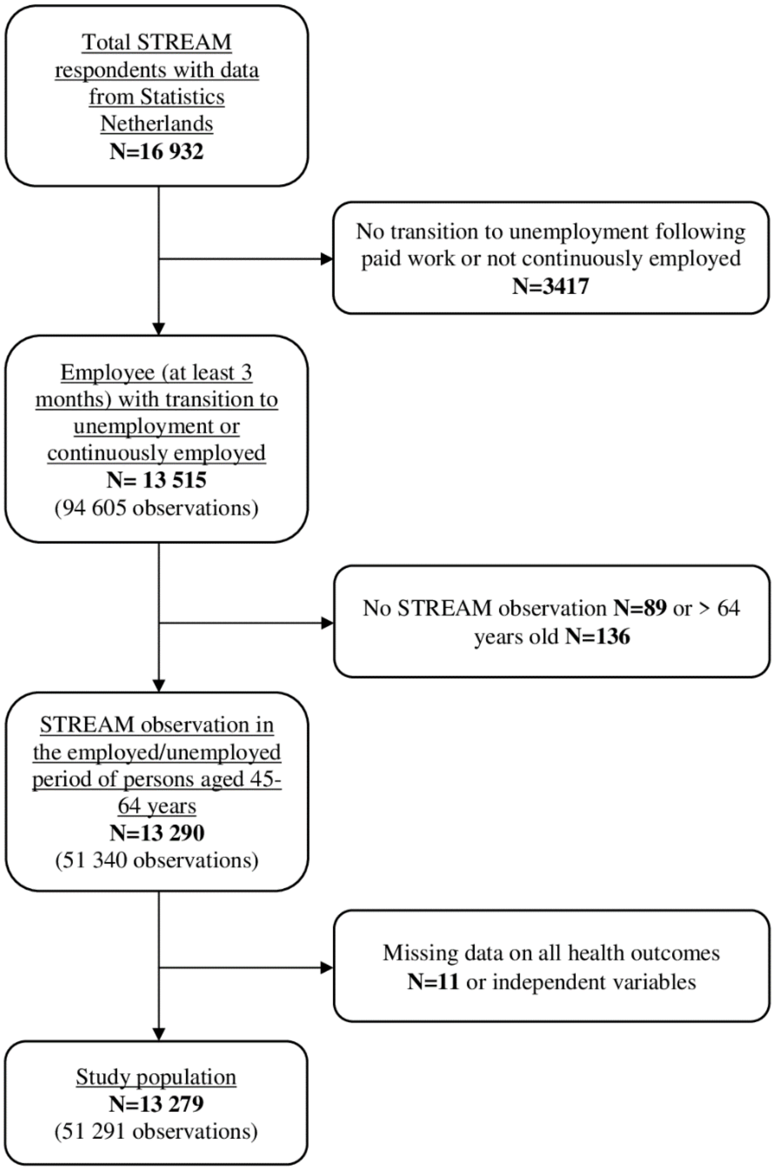

Figure S1. Flowchart presenting the selection of the study population for the analysis on the transition from paid work to unemployment

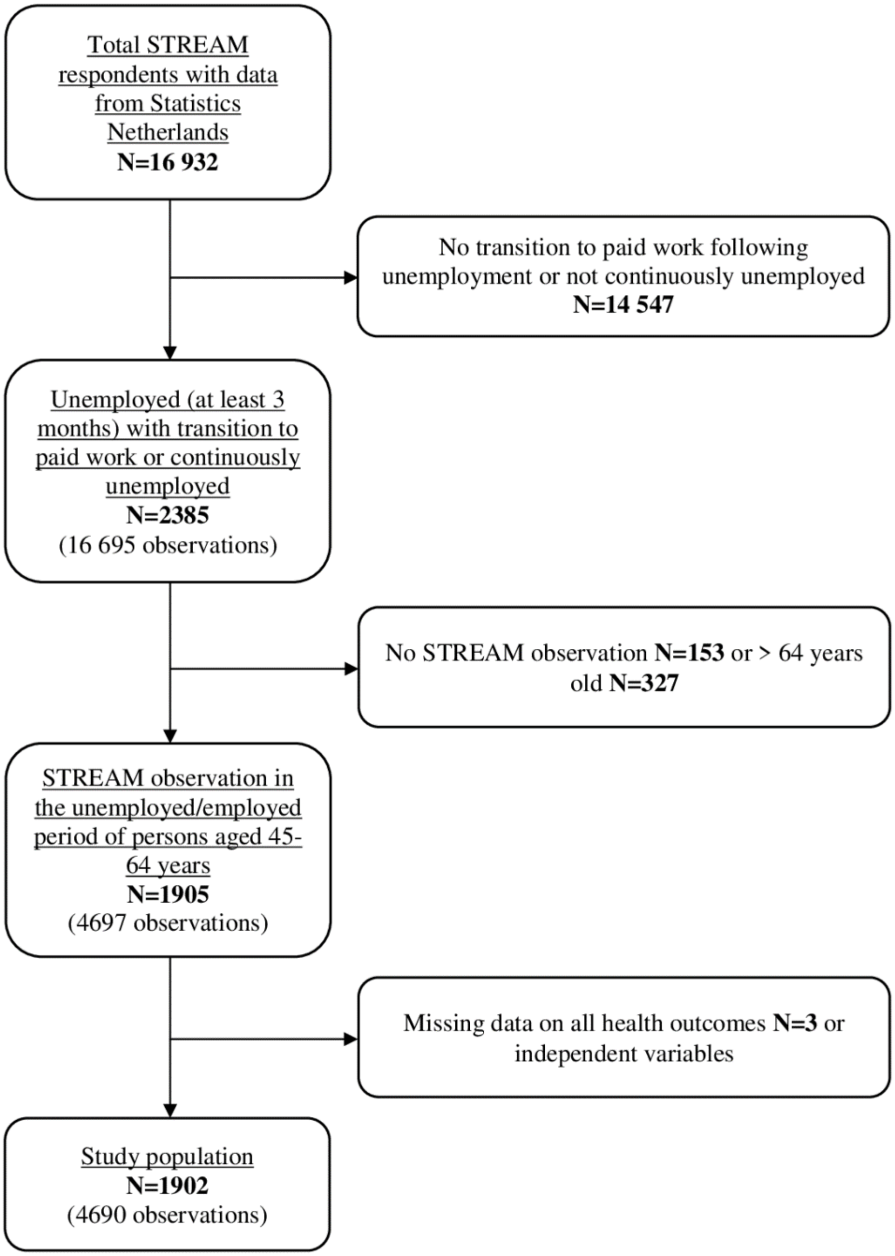

Figure S2. Flowchart presenting the selection of the study population for the analysis on the transition from unemployment to paid work

Table S1. Changes in mental health among Dutch persons aged 45–64 years in the years before, during, and after the transition from paid employment to unemployment and differences between unfavorable and favorable/neutral working conditions

	Mental health (0-100)		
	Psychological job demands	Autonomy	Physical workload
	b (95% CI) [a]	b (95% CI) [a]	b (95% CI) [a]
Transition from paid employment to unemployment			
Before transition			
Difference between unfavorable and favorable/neutral working conditions	**-1.82** (-2.05– -1.59)	**-1.69** (-2.20– -1.18)	**-1.47** (-1.97– -0.97)
Difference between persons becoming unemployed & continuously employed persons	**-2.44** (-2.90– -1.98)	**-2.40** (-2.86– -1.94)	**-2.46** (-2.92– -2.00)
Annual change among continuously employed persons	-0.01 (-0.04–0.01)	-0.01 (-0.04–0.02)	-0.01 (-0.04–0.01)
Annual change among persons becoming unemployed			
Unfavorable working conditions	**-0.51** [b c] (-0.70– -0.31)	**-0.45** [b] (-0.83– -0.06)	0.08 (-0.42–0.57)
Favorable/neutral working conditions	**-0.20** [b] (-0.37– -0.04)	**-0.31** [b] (-0.45– -0.17)	**-0.34** [b] (-0.48– -0.20)
During transition			
Short-term change in the year of becoming unemployed			
Unfavorable working conditions	**2.68** [c] (1.70–3.66)	**3.72** [c] (1.72–5.72)	1.70 (-0.81–4.20)
Favorable/neutral working conditions	0.60 (-0.14–1.35)	**1.18** (0.54–1.82)	**1.40** (0.77–2.03)
After transition			
Annual change among continuously employed persons	-0.01 (-0.04–0.01)	-0.01 (-0.04–0.02)	-0.01 (-0.04–0.01)
Annual change among persons who became unemployed			
Unfavorable working conditions	0.19 (-0.39–0.77)	-0.92 [c] (-1.99–0.14)	-1.42 [c] (-2.89–0.04)
Favorable/neutral working conditions	**0.50** (0.13–0.86)	**0.50** (0.18–0.82)	**0.46** (0.15–0.78)
N	13 103	13 123	13 112

Bold: estimate is statistically significant at the 0.05 level

[a] Associations are adjusted for age, gender and educational level

[b] Annual change among persons becoming unemployed is significantly different compared to persons who do not change in work status at the 0.05 level

[c] Difference between persons with unfavorable working conditions is significantly different compared to persons with favorable or neutral working conditions at the 0.05 level

Table S2. Changes in BMI among Dutch persons aged 45–64 years in the years before, during, and after the transition from paid employment to unemployment and differences between unfavorable and favorable/neutral working conditions

	BMI (kg/m²)		
	Psychological job demands	Autonomy	Physical workload
	b (95% CI) [a]	b (95% CI) [a]	b (95% CI) [a]
Transition from paid employment to unemployment			
Before transition			
Difference between unfavorable and favorable/neutral working conditions	-0.11 (-0.27–0.04)	-0.02 (-0.35–0.31)	-0.16 (-0.49–0.17)
Difference between persons becoming unemployed & continuously employed persons	**0.45** (0.20–0.70)	**0.45** (0.21–0.70)	**0.45** (0.20–0.70)
Annual change among continuously employed persons	**0.04** (0.03–0.04)	**0.04** (0.03–0.04)	**0.04** (0.03–0.04)
Annual change among persons becoming unemployed			
Unfavorable working conditions	**0.12** [b] (0.06–0.18)	0.08 (-0.04–0.20)	**0.28** [b c] (0.12–0.44)
Favorable/neutral working conditions	**0.07** (0.03–0.12)	**0.09** [b] (0.06–0.13)	**0.08** [b] (0.04–0.12)
During transition			
Short-term change in the year of becoming unemployed			
Unfavorable working conditions	-0.03 (-0.30–0.24)	-0.11 (-0.67–0.46)	-0.32 (-1.03–0.39)
Favorable/neutral working conditions	-0.15 (-0.35–0.05)	-0.11 (-0.28–0.06)	-0.09 (-0.26–0.07)
After transition			
Annual change among continuously employed persons	**0.04** (0.03–0.04)	**0.04** (0.03–0.04)	**0.04** (0.03–0.04)
Annual change among persons who became unemployed			
Unfavorable working conditions	0.09 (-0.06–0.25)	**0.44** [c] (0.16–0.72)	0.18 (-0.20–0.57)
Favorable/neutral working conditions	**0.12** (0.03–0.22)	0.08 (-0.01–0.16)	**0.11** (0.02–0.19)
N	13 105	13 126	13 115

Bold: estimate is statistically significant at the 0.05 level

[a] Associations are adjusted for age, gender and educational level

[b] Annual change among persons becoming unemployed is significantly different compared to persons who do not change in work status at the 0.05 level

[c] Difference between persons with unfavorable working conditions is significantly different compared to persons with favorable or neutral working conditions at the 0.05 level

Table S3. Changes in mental health among Dutch persons aged 45–64 years in the years before, during, and after the transition from unemployment to paid work and differences between unfavorable and favorable/neutral working conditions

	Mental health (0-100)		
	Psychological job demands	Autonomy	Physical workload
	b (95% CI) [a]	b (95% CI) [a]	b (95% CI) [a]
Transition from unemployment to paid work			
Before transition			
Difference between persons becoming employed & continuously unemployed persons	**3.65** (2.58–4.72)	**3.67** (2.60–4.74)	**3.66** (2.59–4.73)
Annual change among continuously unemployed persons	**0.34** (0.21–0.47)	**0.34** (0.21–0.47)	**0.34** (0.21–0.47)
Annual change among persons becoming employed	0.42 (-0.21–1.05)	0.44 (-0.19–1.06)	0.43 (-0.20–1.05)
During transition			
Short-term change in the year of becoming employed			
Unfavorable working conditions	0.14 (-1.31–1.59)	0.59 (-1.87–3.04)	0.72 (-2.00–3.43)
Favorable/neutral working conditions	1.15 (-0.01–2.31)	0.77 (-0.28–1.82)	0.80 (-0.24–1.85)
After transition			
Annual change among continuously unemployed persons	**0.34** (0.21–0.47)	**0.34** (0.21–0.47)	**0.34** (0.21–0.47)
Annual change among persons who became employed			
Unfavorable working conditions	-0.23 (-0.72–0.27)	0.21 (-0.92–1.34)	-0.61 (-1.59–0.36)
Favorable/neutral working conditions	-0.22 (-0.58–0.14)	-0.25 (-0.55–0.05)	-0.18 (-0.49–0.12)
N	1877	1877	1877

Bold: estimate is statistically significant at the 0.05 level

[a] Associations are adjusted for age, gender and educational level

Table S4. Changes in BMI among Dutch persons aged 45–64 years in the years before, during, and after the transition from unemployment to paid work and differences between unfavorable and favorable/neutral working conditions

	BMI (kg/m²)		
	Psychological job demands	Autonomy	Physical workload
	b (95% CI) [a]	b (95% CI) [a]	b (95% CI) [a]
Transition from unemployment to paid work			
Before transition			
Difference between persons becoming employed & continuously unemployed persons	**-0.62** (-1.15– -0.10)	**-0.62** (-1.14– -0.10)	**-0.63** (-1.15– -0.11)
Annual change among continuously unemployed persons	**0.05** (0.00–0.09)	**0.05** (0.00–0.09)	**0.05** (0.00–0.09)
Annual change among persons becoming employed	-0.13 (-0.32–0.05)	-0.13 (-0.31–0.05)	-0.14 (-0.32–0.04)
During transition			
Short-term change in the year of becoming employed			
Unfavorable working conditions	-0.32 (-0.76–0.11)	0.08 (-0.66–0.83)	-0.65 (-1.48–0.17)
Favorable/neutral working conditions	-0.04 (-0.38–0.30)	-0.17 (-0.47–0.13)	-0.08 (-0.38–0.21)
After transition			
Annual change among continuously unemployed persons	**0.05** (0.00–0.09)	**0.05** (0.00–0.09)	**0.05** (0.00–0.09)
Annual change among persons who became employed			
Unfavorable working conditions	**0.16** (0.02–0.30)	0.28 (-0.04–0.60)	0.12 (-0.16–0.39)
Favorable/neutral working conditions	0.10 (-0.00–0.20)	**0.11** (0.03–0.20)	**0.12** (0.04–0.21)
N	1880	1880	1880

Bold: estimate is statistically significant at the 0.05 level

[a] Associations are adjusted for age, gender and educational level
